# Serial MTJ-Based TMR Sensors in Bridge Configuration for Detection of Fractured Steel Bar in Magnetic Flux Leakage Testing

**DOI:** 10.3390/s21020668

**Published:** 2021-01-19

**Authors:** Zhenhu Jin, Muhamad Arif Ihsan Mohd Noor Sam, Mikihiko Oogane, Yasuo Ando

**Affiliations:** 1 Department of Applied Physics, Tohoku University, Sendai 980-8579, Japan; muhamad.arif.ihsan.bin.mohd.noor.sam.s5@dc.tohoku.ac.jp (M.A.I.M.N.S.); oogane@mlab.apph.tohoku.ac.jp (M.O.); ando@mlab.apph.tohoku.ac.jp (Y.A.); 2 Graduate Program in Spintronics, Tohoku University, Sendai 980-8578, Japan; 3 Center for Science and Innovation in Spintronics (Core Research Cluster), Organization for Advanced Studies, Tohoku University, Sendai 980-8577, Japan; 4 Center for Spintronics Research Network, Tohoku University, Sendai 980-8577, Japan

**Keywords:** nondestructive testing, magnetic flux leakage testing, magnetoresistive sensor, tunnel magnetoresistance sensor, magnetic tunnel junctions

## Abstract

Thanks to high sensitivity, excellent scalability, and low power consumption, magnetic tunnel junction (MTJ)-based tunnel magnetoresistance (TMR) sensors have been widely implemented in various industrial fields. In nondestructive magnetic flux leakage testing, the magnetic sensor plays a significant role in the detection results. As highly sensitive sensors, integrated MTJs can suppress frequency-dependent noise and thereby decrease detectivity; therefore, serial MTJ-based sensors allow for the design of high-performance sensors to measure variations in magnetic fields. In the present work, we fabricated serial MTJ-based TMR sensors and connected them to a full Wheatstone bridge circuit. Because noise power can be suppressed by using bridge configuration, the TMR sensor with Wheatstone bridge configuration showed low noise spectral density (0.19 μV/Hz^0.5^) and excellent detectivity (5.29 × 10^−8^ Oe/Hz^0.5^) at a frequency of 1 Hz. Furthermore, in magnetic flux leakage testing, compared with one TMR sensor, the Wheatstone bridge TMR sensors provided a higher signal-to-noise ratio for inspection of a steel bar. The one TMR sensor system could provide a high defect signal due to its high sensitivity at low lift-off (4 cm). However, as a result of its excellent detectivity, the full Wheatstone bridge-based TMR sensor detected the defect even at high lift-off (20 cm). This suggests that the developed TMR sensor provides excellent detectivity, detecting weak field changes in magnetic flux leakage testing.

## 1. Introduction

It is well known that nondestructive testing (NDT) plays an increasingly important role in various industrial fields in inspecting or evaluating various materials, components, or assemblies without causing damage to the test object. Frequently used NDT techniques are visual, ultrasonic, radiographic, liquid penetrant, and electromagnetic testing. As an electromagnetic NDT technique, magnetic flux leakage (MFL) testing is widely utilized to examine ferromagnetic objects, such as steel bars, pipelines, and wire ropes, in many industrial fields [1,2,3,4]. In particular, for inspection concrete steel bar, traditional NDT methods such as visual or magnetic particle testing is difficult to be used due to the effect of concrete shielding. Therefore, as a noncontact, fast, and reliable NDT method, MFL testing is mostly used for quality control and locating defects. In MFL testing, powerful magnets or excitation coils are often used to magnetize ferromagnetic objects, and then magnetic lines of flux flow through the object and form a magnetic path between poles. When a flawless ferromagnetic object is inspected, the magnetic lines of flux are contained within it and are difficult to detect in the air surrounding the object. If a defect, such as a fracture, crack, or corrosion, is present in the steel bar, its magnetic permeability is drastically changed, and the distribution of magnetic flux leakage surrounding the defect is inevitably disturbed. By measuring the variations in the magnetic field, the defect properties, such as size and location, can be determined, and the ferromagnetic object can thereby be inspected using MFL testing. The application of magnetic sensors is imperative in the inspection of objects in MFL testing. Since an inhomogeneous magnetic flux leakage field exists around the defect location, the magnetic sensor must be highly sensitive to variations in magnetic fields to determine tiny defects during MFL testing [5,6]. Based on a highly sensitive sensor, obtaining the spatial distribution of the magnetic flux leakage field is essential to accurately estimate the location or dimensional characteristics of defects [7,8].

Currently, induction coil sensors are frequently used in conventional MFL testing [9]. Their sensitivity is largely dependent on their size characteristics and field frequency. Improving the defect signal requires increasing the coil number and size, which inevitably results in increasing the device size and decreasing the spatial resolution for MFL testing [2,10]. Several studies reported that Hall-effect-based sensors could detect surface-breaking defects in MFL testing [11,12,13]. Because of the low cost, low power consumption, and excellent stability of the frequency response, the Hall sensor is considered to be a good choice as a magnetic sensor in electromagnetic NDT applications. However, the sensitivity limit makes it difficult to detect small magnetic field changes and identify the size and location of tiny defects. Other sensors, such as optically pumped magnetometers and superconducting quantum interference devices (SQUIDs), are highly sensitive to the weak magnetic fields on the order of sub-picotesla. Based on the Josephson effect, a SQUID containing Josephson junctions showed low noise characteristics and field detectivity of 50 fT/Hz^0.5^ at frequencies as low as 10 Hz [14]. However, the high cost and ultralow operation temperature are extremely critical barriers to further application.

After the discovery of high magnetoresistance (MR) in ferromagnetic multilayers [15], MR effect-based sensors, such as anisotropic magnetoresistance (AMR), giant magnetoresistance (GMR), and tunneling magnetoresistance (TMR) sensors, have been widely investigated for the detection of small magnetic fields in various applications [16,17,18]. Among these MR sensors, magnetic tunnel junction (MTJ)-based TMR sensors have attracted much attention due to their high MR ratio at room temperature. The MTJ structure consists of two ferromagnetic layers separated by an insulating layer. Its electrical resistance is determined by the relative orientation of magnetic moments in the two ferromagnetic layers. An MTJ with the parallel alignment of the magnetic moment has lower resistance, whereas in the opposite case—one with the anti-parallel alignment of the magnetic moment—the MTJ has a higher resistance. If an external magnetic field is applied to the MTJ and one of the ferromagnetic layers is more easily magnetized than the other, the external magnetic field can be evaluated by measuring the resistance in the MTJ. Some studies showed that a crystalline MgO-barrier-based MTJ exhibited over 200% MR ratio at room temperature [19,20]. Based on the crystalline barrier, previous studies demonstrated that optimized MTJs with synthetic antiferromagnetic-free layers could provide high sensitivity to sensing the variations in external magnetic fields [21]. Moreover, thanks to excellent miniaturization, MTJs can be fabricated for submicrometer-sized devices and connected in a series, which can enhance detectivity to obtain a high signal-to-noise ratio (SNR) [22,23]. These study results suggest that developed MTJs enable us to design sensors with high sensitivity and high spatial resolution for MFL testing applications.

To date, many studies have focused on optimizing the magnetic multilayer structure or configuration of integrated MTJs. Serval approaches, such as selecting ferromagnetic material with high spin polarization, optimizing the interface between ferromagnetic and insulating layers, decreasing the anisotropy field of the ferromagnetic layer, and developing a high-gain magnetic flux concentrator (MFC), were utilized to further enhance the sensitivity of TMR sensors [24]. However, MTJs connected in a Wheatstone bridge circuit can effectively eliminate the output fluctuation caused by temperature drift, which improves sensor performance in electromagnetic NDT [25,26]. Hence, in the present work, we fabricated and characterized a serial MTJ-based TMR sensor and four serial MTJ-based TMR sensors connected in a full Wheatstone bridge circuit (FWB-TMR sensor). Their output, sensitivity, and noise spectral density were investigated to determine their magnetic field-sensing capability. Furthermore, the sensors were used to inspect steel bars in MFL testing, and then their output signals and SNR at various lift-off heights were investigated to determine their performance for MFL testing.

## 2. Materials and Processes

**Figure 1:**
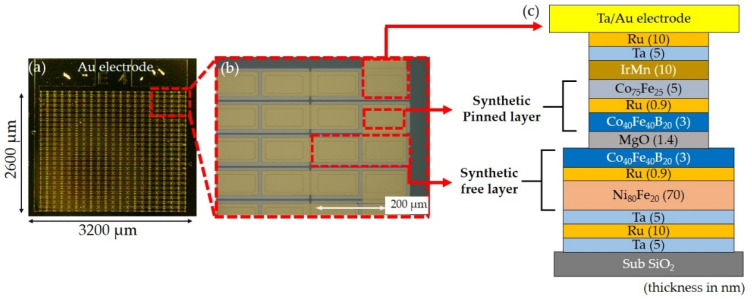
(**a**,**b**) Microscopy image of a tunnel magnetoresistance (TMR) sensor containing 500 magnetic tunnel junctions (MTJs) in series. The two pinned junctions were etched onto the bottom electrode of the free layer. (**c**) Stacking structure of MTJ film.

The MTJ magnetic films were prepared with the use of an ultrahigh vacuum sputtering system (*P*_base_ < 3 × 10^–6^ Pa), and the stacking structure of films was SiO_2_/Ta(5)/Ru(10)/Ta(5)/Ni_80_Fe_20_(70)/Ru(0.9)/Co_40_Fe_40_B_20_(3)/MgO(1.4)/Co_40_Fe_40_B_20_(3)/Ru(0.9)/Co_75_Fe_25_(5)/Ir_22_Mn_78_(10)/Ta(8) (values in parentheses denote the thickness in nanometers). The crystallized interface of CoFeB/MgO can result in a high TMR ratio due to coherent Δ_1_ band tunneling [27,28]. Furthermore, the NiFe/Ru/CoFeB tri-layer was deposited as a synthetic antiferromagnetic-free (SAF) layer, and NiFe and CoFeB layers were antiferromagnetically coupled by a thin Ru layer. The thick 70 nm NiFe layer exhibits a low magnetic anisotropy field, and thus the magnetization reversal of the SAF layer is sensitive to variations in applied magnetic fields along the pinning direction. The serial MTJs were fabricated using photolithography and argon ion milling. During the fabrication process, the first milling was performed for the fabrication of isolated SAF layer patterns, and the second milling was stopped at the MgO layer; therefore, the SAF layer patterns resulted in continuous electrodes. To separate the bottom and upper electrodes, the 200 nm thick SiO_2_ layer was deposited by chemical vapor deposition (CVD). Furthermore, the contact path was etched by ion milling for each junction and the upper Au electrode was deposited so that the current could flow through all junctions. Figure 1 exhibits the 500 pinned junctions that were connected in series, each junction with an area of 140 × 80 μm^2^.

After microfabrication, two successive annealing processes were used to improve the magnetoresistance and linear response. The fabricated sensors were annealed at 350 °C while an external field of 10 k Oe was applied along the easy axis of the free layer pattern. After the first annealing step, the tunneling magnetoresistance was significantly increased due to crystalline CoFeB and MgO layers in the MTJs. The second annealing step was performed at 300 °C while the external field (10 kOe) was applied along the hard axis of the free layer pattern. After the second annealing, the pinning direction was orthogonal to the easy axis of the free layer, which resulted in linear response to the applied magnetic field for the MTJs [21].

**Figure 2:**
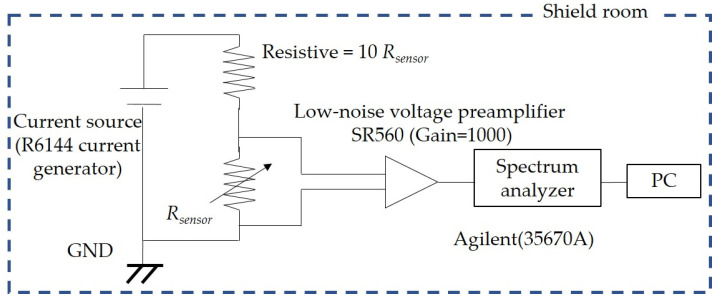
Schematic diagram of the measurement system.

Additionally, to determine the noise characteristics of sensors, a measurement system was utilized to measure the noise spectral density. Figure 2 depicts a schematic diagram of the noise measurement system. These measurements were carried out in a magnetically shielded room that provided a low-magnetic-field environment. A low noise voltage preamplifier (SR560) with a gain of 1000 was used to maximize the outputs, and a spectrum analyzer (35670A) was used to determine the noise spectral density.

**Figure 3:**
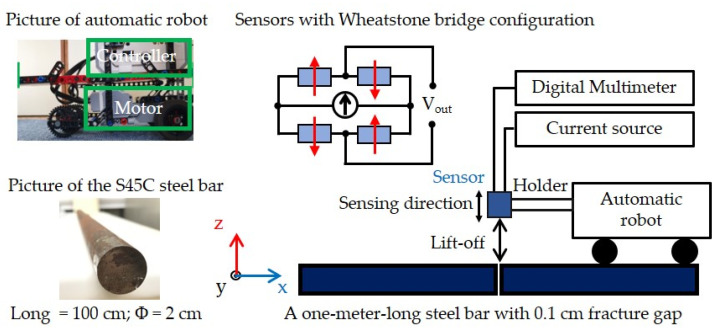
Schematic diagram of the developed magnetic flux leakage (MFL) testing system.

Before MFL testing, the steel bars were magnetized using a solenoid consisting of an 875-turn coil with a length of 1.44 m. A current of 2.0 A was passed through the solenoid, creating a magnetic field of approximately 15.24 Oe. Figure 3 depicts a schematic diagram of the automated MFL testing system and inspected object information in the present study. The system consists of a current source, a digital multimeter, and an automatic robot with fabricated TMR sensors. Considering the presence of a background magnetic field, measuring the *z*-axis component of the MFL field *B* was less affected by the background magnetic field when sensing variations compared to when measuring the *x*-axis component of *B* [29]. Therefore, the sensing direction (pinning direction) of the sensor was set parallel to the *z*-axis field, which was sensitive to changes in the *z*-axis component of magnetic fields. A flawless and a fractured steel bar (S45C) with a 0.1 cm fracture gap were prepared for MFL testing, respectively. The scanning was carried out with a step of 1 mm, and its length was set to 100 cm along the long direction (*x*-axis direction) of the steel bar. The output voltage was recorded in real-time so that the sensor can timely detect changes in the magnetic field distribution. In addition, the automatic robot was used to move the TMR sensor along the *x*-axis direction (long direction of the steel bar) to measure the magnetic flux signal, and the motor was equipped with a magnetic shield for suppression of impact from the motor. The lift-off (distance between the sensor and the steel bar surface) was manually adjusted along the *z*-axis from 4 to 20 cm. During MFL testing, the current source provided an applied current of 1 mA to the sensor, and the digital multimeter recorded the output voltage of the sensor.

## 3. Results and Discussion

### 3.1. Characterization of TMR Sensor

**Figure 4:**
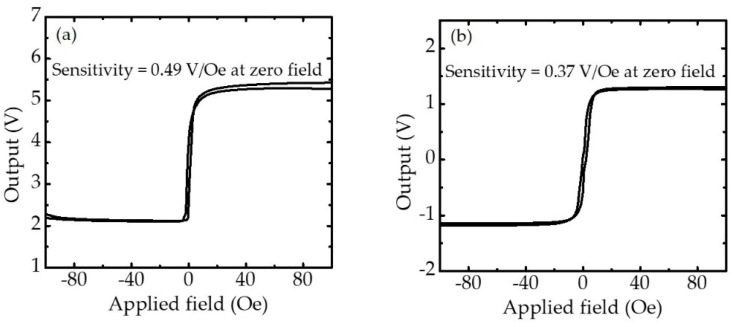
(**a**) Outputs for one serial MTJ sensor and (**b**) four serial MTJ sensors connected in a full Wheatstone bridge circuit at room temperature. Sensitivity is determined by Δ*V*/Δ*H* term slope at zero field. The linear range is defined as a dynamic range with nonlinearity of 10% FS for each sensor.

Magnetoresistance transfer curves were measured at room temperature to determine sensor performance. Figure 4 shows the outputs of one serial MTJ sensor and four serial MTJ sensors connected in a Wheatstone bridge circuit with a bias current of 0.7 mA when the external field was in the range of 100 to −100 Oe. The MTJ stacks showed an approximate *R_p_A* (resistance in the parallel magnetization state × junction area) value of (5.62 ± 0.53) × 10^4^ Ωμm^2^. The two resistance states and linear range were observed in the transfer curves of the one-TMR and FWB-TMR sensors when the external field was swept from –100 to +100 Oe and back to +100 to −100 Oe. For one serial MTJ sensor, the linear transfer curves (−2 to ~6 Oe) and sensitivity of 0.49 V/Oe were obtained around zero fields. For the sensor with Wheatstone bridge configuration, four TMR sensors with highly similar resistance values (2532 ± 240 Ω) were connected in a full Wheatstone circuit. Although there was a small imbalance in sensor resistance due to the slightly different *R_p_A* value, an external resistor was connected to each TMR sensor so that the resistance imbalance was compensated and the voltage offset decreased. Consequently, the sensitivity decreased to 0.37 V/Oe and the linear range increased to ±6 Oe.

**Figure 5:**
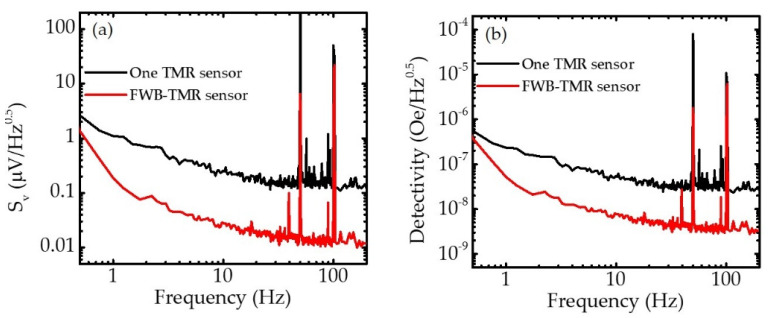
(**a**) Noise spectral density *S_v_* as a function of frequency for one serial MTJ sensor and FWB-TMR sensor with bias current of 0.7 mA at zero external field. (**b**) Detectivity for one serial MTJ sensor and FWB-TMR sensor at zero external field.

Furthermore, the noise spectral density *S*_v_ was characterized at zero field. Figure 5a shows the *S*_v_ of one serial MTJ sensor and FWB-TMR sensor. The noise of an MTJ contains several factors: amplifier, thermal-shot, electrical 1/*f*, and magnetic 1/*f* noise. Here, the electrical and magnetic 1/*f* noises dominate only in the low-frequency regime, whereas the thermal-shot noise as white noise presents in both the low- and high-frequency regimes. The result showed the full FWB-TMR sensor exhibited a considerably lower *S*_v_ value because the use of a bridge configuration can significantly reduce the thermal drift and the thermal-shot noise [30,31]. Since the four TMR sensors were arranged in a Wheatstone bridge configuration, the output voltage is zero at zero magnetic field, and the resistance and thermal noise for the individual sensors could be canceled in the output voltage of the FWB-TMR sensor. Consequently, compared with one serial MTJ sensor, the FWB-TMR sensor showed lower noise voltage. The detectivity *D* is defined as:

$$D=S_{v}/(\Delta V/\Delta H)$$

where *S_v_* is the noise term and Δ*V/*Δ*H* is the sensitivity term. As an important characteristic of the sensor, *D* is the minimum detectable field determined by noise and sensitivity. Figure 5b shows the *D* of sensors at zero field. Although the one TMR sensor showed higher sensitivity, the FWB-TMR sensor showed considerably low detectivity due to the low noise voltage. This result indicated that the four serial MTJ sensors connected in a full Wheatstone circuit provided a high SNR during MFL testing.

### 3.2. MFL Testing with TMR Sensors 

**Figure 6:**
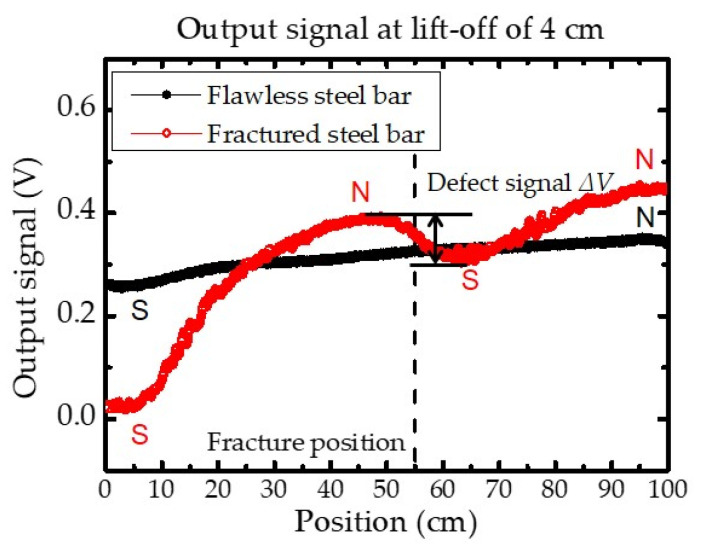
MFL testing results of flawless and fractured steel bars for one serial MTJ sensor. The peak-to-valley amplitude of the output signal around the fracture position is defined as defect signal Δ*V*. The estimated magnet’s north and south poles are labeled with “N” and “S”. Estimated defect location is defined as the center of the peak to valley along the *x*-axis direction. The dotted line represents the actual fracture position on the *x*-axis.

To verify the performance of the constructed MFL testing system, the one serial MTJ sensor was used to inspect a flawless and a fractured steel bar (0.1 cm gap), respectively. Figure 6 demonstrates the scan result that the z-component of magnetic flux leakage was measured, and the output signal corresponding to the change in magnetic fields. For the inspection of the flawless steel bar, since the magnetic poles induced in the magnetized steel bar, the output signal variation appeared during the scan from the left (position = 0 cm) to the right (position = 100 cm) along the *x*-axis. In comparison, the obvious variation appeared in the output signal when the sensor was above the fracture position (position = 55 cm). Considering that the presence of a fracture caused the magnetic flux lines to leak out from the fracture as well as magnetic poles in the steel bars, a significant disturbance of the magnetic flux intensity occurred surrounding the fracture location. Consequently, the variation was obtained between the peak and trough (Δ*V*) in the output signal, and the fracture position was determined.

**Figure 7:**
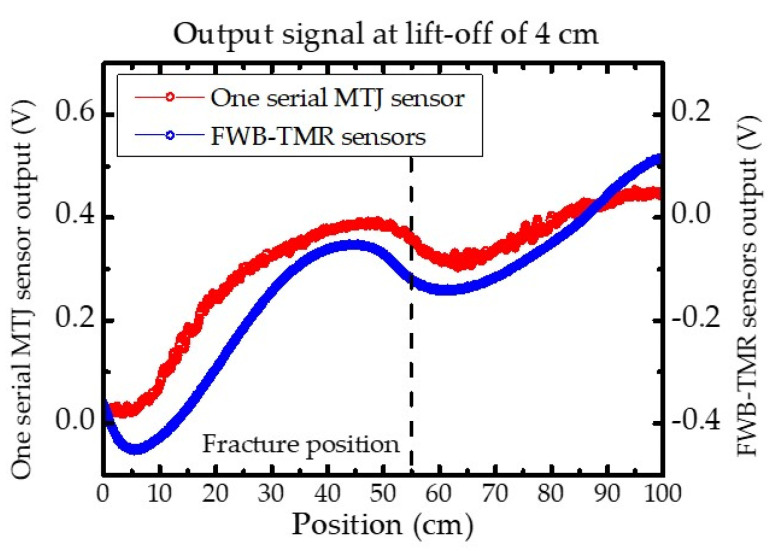
MFL testing results of fractured steel bars for one serial MTJ sensor and FWB-TMR sensor. The Δ*V* of 0.93 V and 0.87 V were obtained by using one serial MTJ sensor and FWB-TMR sensor, respectively. The dotted line represents the actual fracture position on the *x*-axis.

Figure 7 demonstrates the one serial MTJ sensor and the FWB-TMR sensor provided different outputs during the inspection of the fractured steel bars, respectively. Since the inspection result depends on the sensitivity of the sensor, the Δ*V* of the one TMR sensor was a little higher than that of the FWB-TMR sensors due to high sensitivity. However, the noise amplitude in the output signal of one TMR sensor was higher than the one of the FWB-TMR sensors. Since the noise amplitude depends on the noise characteristics of sensors, the high noise spectral density certainly caused a high noise amplitude and resultant low SNR for detection of a defect in the steel bar.

**Figure 8:**
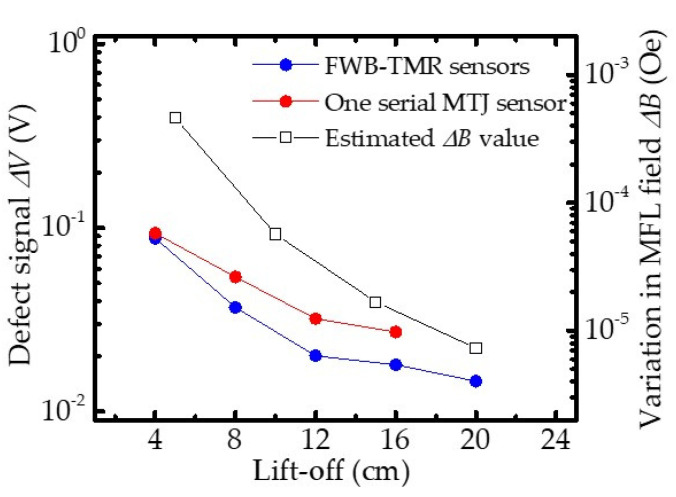
Dependence of defect signal Δ*V* and variations in estimated MFL field Δ*B_z_* on various lift-off values.

Furthermore, theoretically, a higher lift-off value reduces Δ*B* due to the inhomogeneous magnetic field around the inspected steel bar. According to the magnetic dipole theory, the calculated Δ*B_z_* (the *z*-axis component of the MFL field) should strongly depend on the lift-off value [6,32]. Figure 8 shows that the Δ*V* of sensors and estimated Δ*B_z_* decreased with increasing lift-off. This indicates that the dependence between Δ*V* and lift-off value agrees well with the theoretical predictions that the estimated Δ*B* decreases as the lift-off value increases. Moreover, thanks to the high sensitivity, the one TMR sensor exhibited higher Δ*V* values in detecting Δ*B* with various lift-off values compared with the FWB-TMR sensor. However, due to the higher noise voltage, the measurement with one serial MTJ sensor failed to obtain Δ*V* after lift-off of 16 cm, whereas Δ*V* was obtained by the FWB-TMR sensor. As a result of the clear Δ*V* in the output, the fracture could be located by the FWB-TMR sensor even at lift-off of 20 cm.

**Figure 9:**
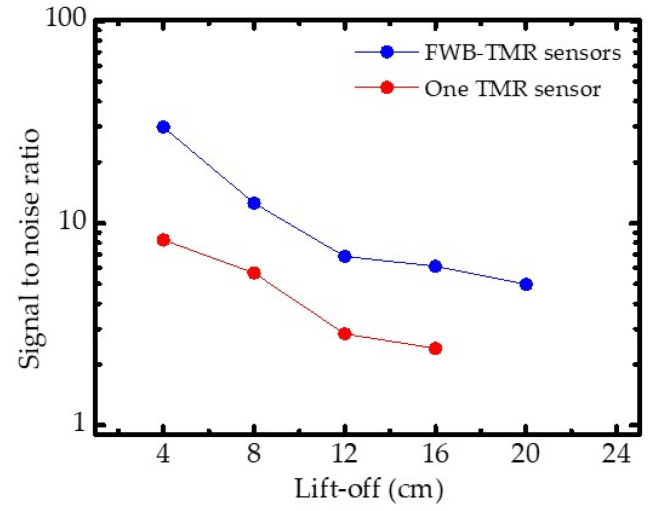
Dependence of SNR of defect detection on various lift-off values.

Figure 9 shows the dependence of SNR on the lift-off when detecting a fracture with different sensors. When the lift-off was set to 4 cm, both sensors determined the presence of a fracture, providing an SNR of 9 and 30 dB for fracture detection for the serial MTJ and FWB-TMR sensors, respectively. Since Δ*V* strongly depends on the lift-off value in MFL testing, the dependence of SNR values on lift-off is similar to that of Δ*V* on lift-off for both sensors. However, the SNR values were impacted by the background field (earth’s magnetic field) and the noise characteristics of devices in the MFL system. Although the one serial MTJ sensor could detect a minimum field of 10^−6^–10^−7^ Oe at a frequency of 1–10 Hz in a magnetically shielded room, it failed to determine the presence of fracture at lift-off of 20 cm, due to the high noise caused by the background field in the MFL testing system. As the FWB-TMR sensor showed lower noise spectral density and had much better detectivity to sense external fields, the SNR of 4 dB was obtained and the defect presence was successfully determined when the FWB-TMR sensor was used to inspect the fractured steel bar at lift-off of 20 cm. This suggests that the FWB-TMR sensor can detect fracture gap of steel bars in MFL testing at high lift-off.

## 4. Conclusions

In this study, a highly sensitive TMR sensor based on 500 SAF-MTJs connected in series was successfully fabricated for MFL testing of steel bars. Compared with one TMR sensor based on 500 serial MTJs, the four TMR sensors connected in a full Wheatstone bridge circuit (FWB-TMR sensor) showed lower sensitivity due to their nonuniform magnetoresistance. However, a full Wheatstone bridge circuit effectively suppressed the white noise of sensors, so the FWB-TMR sensor provided excellent detectivity of 5.29 × 10^−8^ Oe/Hz^−0.5^ at a frequency of 1 Hz. In MFL testing, both sensors provided defect signals during MFL testing. However, thanks to the low detectivity, a higher SNR was obtained when the gap was detected using the FWB-TMR sensor. The defect signal was obtained and the fracture presence was determined using the FWB-TMR sensor in MFL testing with the lift-off varying from 4 to 20 cm. To detect a tiny defect at high lift-off values in MFL testing, further work will focus on optimizing measurements with low system noise and reducing the influence of the background field. An appropriate magnetization method should be proposed, which could magnetize ferromagnetic objects and reduce the influence of external magnetic fields and system noise during MFL testing. However, this study demonstrated that four highly sensitive TMR sensors connected in a full Wheatstone bridge circuit could be suitable for achieving a high SNR and locating defects in ferromagnetic objects in MFL testing. The present findings are expected to contribute to further NDT applications using TMR sensors.

## Data Availability

All data generated or analyzed during this study are included in this article.
